# Reversible Electrochemical Intercalation and Deintercalation of Fluoride Ions into Host Lattices with Schafarzikite‐Type Structure

**DOI:** 10.1002/open.201800106

**Published:** 2018-08-20

**Authors:** Mohammad Ali Nowroozi, Benjamin de Laune, Oliver Clemens

**Affiliations:** ^1^ Technische Universität Darmstadt Institut für Materialwissenschaft, Fachgebiet Materialdesign durch Synthese Alarich-Weiss-Straße 2 64287 Darmstadt Germany), Fax: (+49) 6151 16 21991; ^2^ School of Chemistry University of Birmingham Birmingham B15 2TT United Kingdom; ^3^ Karlsruher Institut für Technologie (KIT) Institut für Nanotechnologie Hermann-von-Helmholtz-Platz 1 76344 Eggenstein Leopoldshafen Germany

**Keywords:** electrochemical fluorination, fluoride-ion batteries, schafarzikite type structure, topochemical reactions, X-ray diffraction

## Abstract

Herein, we report the successful electrochemical fluorination and defluorination of schafarzikite‐type compounds with the composition Fe_0.5_ m_0.5_Sb_2_O_4_ (M=Mg or Co). We show that electrochemical methods can present a more controllable and less environmentally damaging route for fluorinating compounds in contrast to traditional methods that involve heating samples in F_2_‐rich atmospheres. The reactivity of the host lattices with fluoride during electrochemical fluorination makes this material an interesting candidate for fluoride‐ion battery applications. However, deleterious side reactions with the conductive carbon matrix during fluorination suggests to the contrary. Regardless of the side reactions, the schafarzikite structure was found to be an alternative reversible host lattice for fluoride incorporation and removal in addition to the previously reported Ruddlesden–Popper‐type compounds.

## Graphical abstract

**A gracious host**: Electrochemical fluorination can be considered as an alternative synthesis method for the fluorination of oxides. The advantage of this method compared to conventional chemical routes lies in the safety and controllability, and the intercalation can even be reversed by using this methodology. Herein, the reversible electrochemical fluorination of schafarzikite‐type compounds is reported.

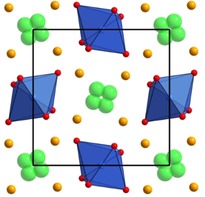

## 1 Introduction

Fluorine insertion into metal oxides has become an interesting topic over the past years, owing to the potential for modifying the electronic,^1^ magnetic,^2^ and superconducting behavior^3^ of host lattices through structural and compositional changes. Furthermore, such oxide materials are considered reversible electrode materials for fluoride‐ion batteries (which were previously based on conversion‐type compounds),^4^ for which currently only compounds with Ruddlesden–Popper‐type structure are known to show principle structural reversibility.^5^

Chemical fluorination of the oxides has predominantly been performed via chemical reactions, for example, by heating samples under flowing F_2_ gas^6^ or with milder fluorination agents such as PVDF.^7^ The use of oxidative agents (F_2_, CuF_2_, AgF_2_) is challenging, and can often lead to the decomposition of the target compounds.^8^ The reason for this originates from the fact that such reagents always work at a certain chemical fluorination potential, which can only be altered by the choice of the metal fluoride. Especially for fluorine gas, the reaction is then mainly controlled by experimental parameters such as temperature, time, and fluorine concentration.6a In recent reports, our group has shown that the electrochemical fluorination of compounds (e.g. LaSrMnO_4_, La_2_CoO_4_, and BaFeO_2.5_) within an all‐solid‐state fluoride‐ion battery can serve as an alternative method^8^, ^9^ for the preparation of oxyfluorides, where the degree of fluorination can be adjusted through tuning by choosing suitable electrochemical potentials and charging times.

**Figure 1:**
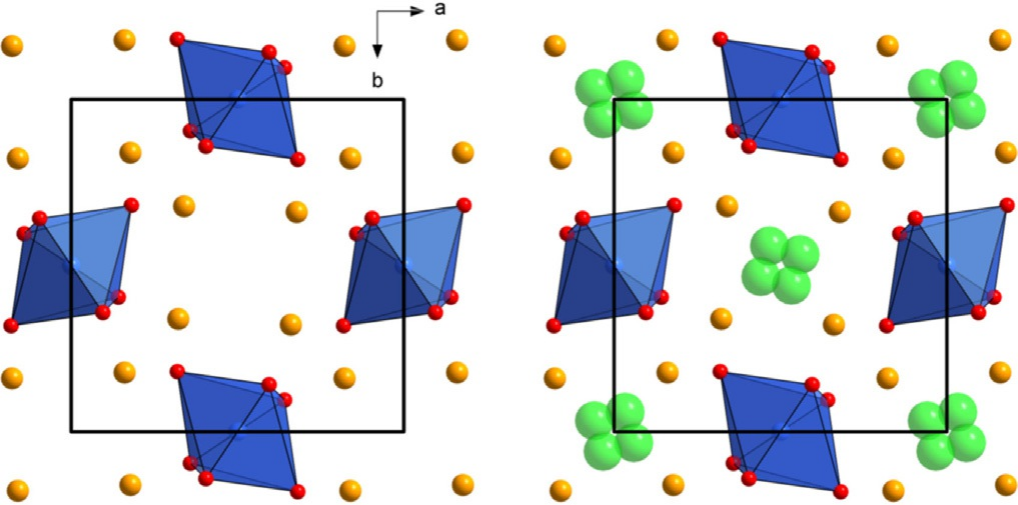
Schematic presentation of non‐fluorinated MSb_2_O_4_ (left) and fluorinated MSb_2_O_4_F_y_ (right) schafarzikite‐type structures. Sb: orange, M (Fe, Co): blue, O: red, F (partially occupied to ca. 15 %): green. Structures are based on data provided in Ref. 6b.

The schafarzikite‐type structure (see Figure 1) of compounds with the composition MSb_2_O_4_ 10 (known for their antiferromagnetic properties with various different magnetic structures)^11^ possesses a tetragonal symmetry (space group *P*4_2_/*mbc*). The structure can be understood as being built up of chains of edge‐linked MO_6_ octahedra running along the [0 0 1] direction; the chains are connected through trigonal pyramidal SbO_3_ units. Recent studies have shown that it is possible to fluorinate variants of this material (see Figure 1), which contain Fe^2+^ on the M site, by using topochemical reactions.6b, 10, 12 The proposed mechanism for fluorination is based on two key principles.6b Firstly, the phase must possess Fe^2+^ to act as the redox active center whilst the degree of oxidation is limited to the amount of Fe^2+^ to be oxidized to Fe^3+^. Furthermore, it has been shown that there is a propensity for the Sb^3+^, which line the walls of the channel, to also play a part in the oxidation process depending on the atmosphere and conditions that the material is heated in.^13^

This material is of interest because of the mechanism for the inclusion of excess fluoride ions within the channel of the structure (see Figure 1). Therefore, it can be considered to be a 1D intercalation material, like olivine‐type materials for lithium‐ion batteries.^14^ This is in contrast to the layered ordering of intercalated fluoride ions within Ruddlesden–Popper‐type compounds, which are 2D intercalation materials (like layered materials, e.g., LiCoO_2_ for lithium‐ion batteries).^15^ For both structure types, intercalated fluoride ions were found to be located on a different crystallographic site than the oxide ions, and such ordering of the intercalated ions is a key feature of intercalation based battery materials.

Here, we build upon the previous study, which explored the chemical fluorination behavior of schafarzikite‐type Mg_0.5_Fe_0.5_Sb_2_O_4_ and Co_0.5_Fe_0.5_Sb_2_O_4_ using gaseous fluorine to form Mg_0.5_Fe_0.5_Sb_2_O_4_F and Co_0.5_Fe_0.5_Sb_2_O_4_F_*x*_ (where *x≈*0.5). In this article, we investigate their suitability for electrochemical applications within all‐solid‐state fluoride‐ion batteries. The inclusion of 0.5 F per formula unit corresponds to the specific charging capacity of roughly 36–39 mA h g^−1^.6b We show that this class of material is found to be the second suitable host material for the fully reversible intercalation/deintercalation of fluoride ions. However, high charging potentials were found to currently impede their use as intercalation‐based cathodes for fluoride‐ion batteries when carbon is used as the conductive additive.

## 2 Results and Discussion

The lattice parameters of the fluoridated samples were obtained from Rietveld analysis of the XRD data9b and can be compared to the parent material and the fluoridated compounds reported previously.^10^ It is necessary to confirm that changes of lattice parameters, which were observed after the charging/discharging of the samples, really resulted from an electrochemical reaction. To verify this, we also investigated fully assembled cells, which were only heated to the battery operation temperature without applying any current. From this, one can rule out unwanted side reactions, which would also result in changes of lattice parameters, for example, an oxide for fluoride substitution reaction with the La_0.9_Ba_0.1_F_2.9_ admixture according to [Eq. (1)]:

(1) $$M{}^{\mathrm{II}}\mathrm{Sb}{}^{\mathrm{III}}{}_{2}O{}_{4}+\mathrm{La}{}_{0.9}\mathrm{Ba}{}_{0.1}F{}_{2.9}\to M{}^{\mathrm{II}}\mathrm{Sb}{}^{\mathrm{III}}{}_{2}O{}_{4-x}F{}_{2x}+\mathrm{La}{}_{0.9}\mathrm{Ba}{}_{0.1}F{}_{2.9-2x}O{}_{x}$$

**Figure 2:**
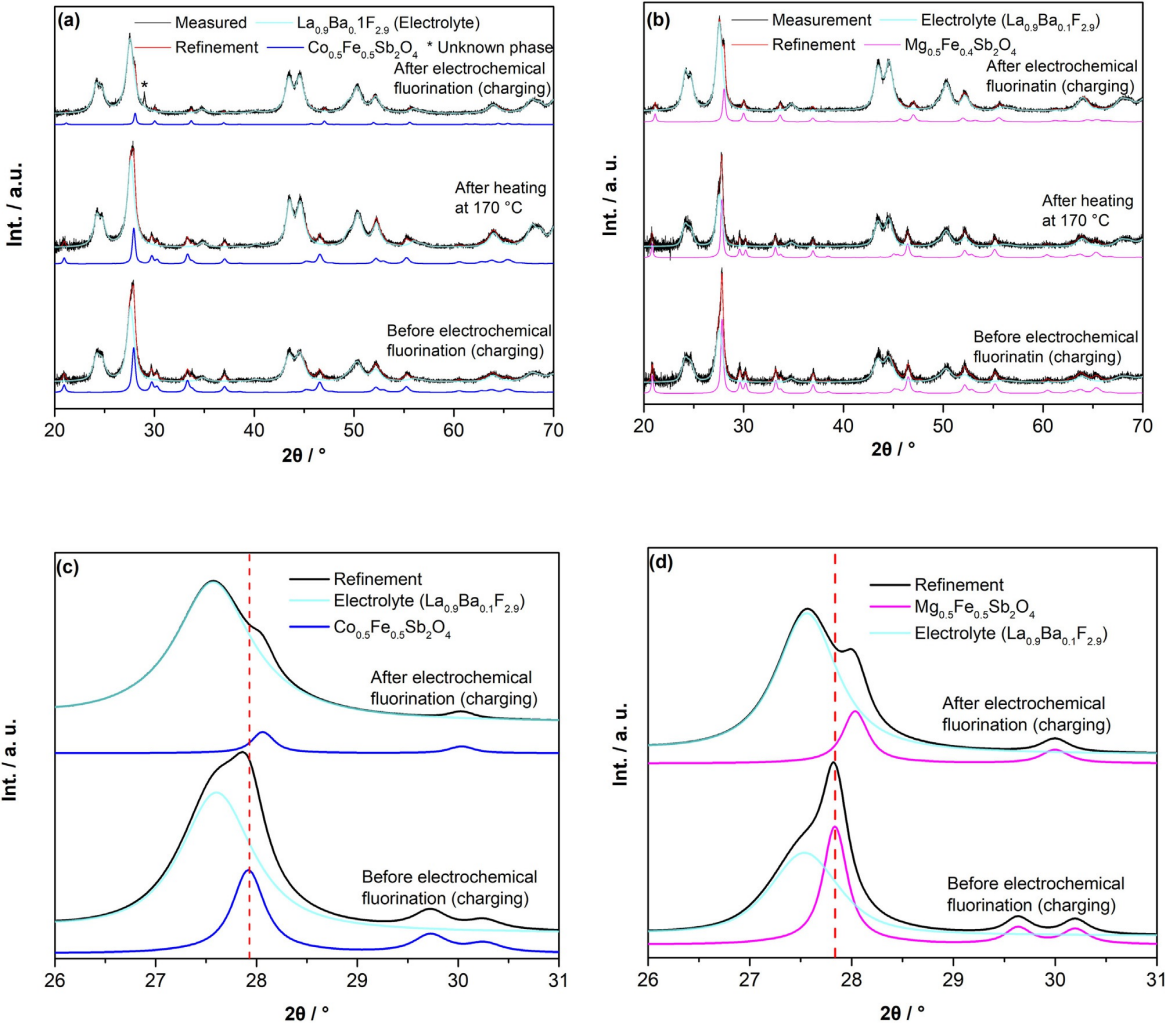
Refined XRD patterns of a) Co_0.5_Fe_0.5_Sb_2_O_4_ and b) Mg_0.5_Fe_0.5_Sb_2_O_4_ before charging, after heating at 170 *°*C, and after charging to 3 V at *T*=170 *°C* and *I*=10 μA (24 μA cm^−2^). Refined XRD patterns of c) Co_0.5_Fe_0.5_Sb_2_O_4_ and d) Mg_0.5_Fe_0.5_Sb_2_O_4_ before charging and after charging to 3 V at *T*=170 *°*C and *I*=10 μA (24 μA cm^−2^).

Indeed, no significant changes of lattice parameters were found after heating at 170 °C for 24 h, confirming the stability of the La_0.9_Ba_0.1_F_2.9_ towards the schafarzikite compounds (Figure 2 a, b and Table 1). This ruled out the possibility for the potential degradation of the parent phase through temperature‐induced non‐oxidative substitution reactions. Hence, all structural changes found on electrochemical treatment can be associated with the electrochemical charging and discharging reactions of the compounds.

**Table 1:** Lattice parameters of Co_0.5_Fe_0.5_Sb_2_O_4_ and Mg_0.5_Fe_0.5_Sb_2_O_4_ (space group *P*4_2_/*mbc*), as observed before and after various electrochemical treatments or heating.

	Co_0.5_Fe_0.5_Sb_2_O_4_			Mg_0.5_Fe_0.5_Sb_2_O_4_		
	*a* [Å]	*c* [Å]	*a*/(c*√2)	*a* [Å]	*c* [Å]	*a*/(c*√2)
Initial material (before milling)	8.5365(3)	5.9302(2)	1.018	8.5420(3)	5.9303(2)	1.018
Within composite mixture (before heating)	8.5469(13)	5.9382(13)	1.018	8.5442(14)	5.9313(12)	1.019
After heating to 170 °C for 24 h	8.5449(12)	5.9383(13)	1.017	8.5520(14)	5.9290(12)	1.020
After electrochemical fluorination (charging)	8.4392(18)	5.9838(28)	0.997	8.4396(13)	5.9766(16)	0.999
After chemical fluorination6b	8.4270(3)	5.9501(2)	1.001	8.4537(6)	5.9451(3)	1.005
After electrochemical defluorination (“forced discharging”)	8.5492(13)	5.9279(13)	1.019	8.5124(11)	5.9423(11)	1.013

**Figure 3:**
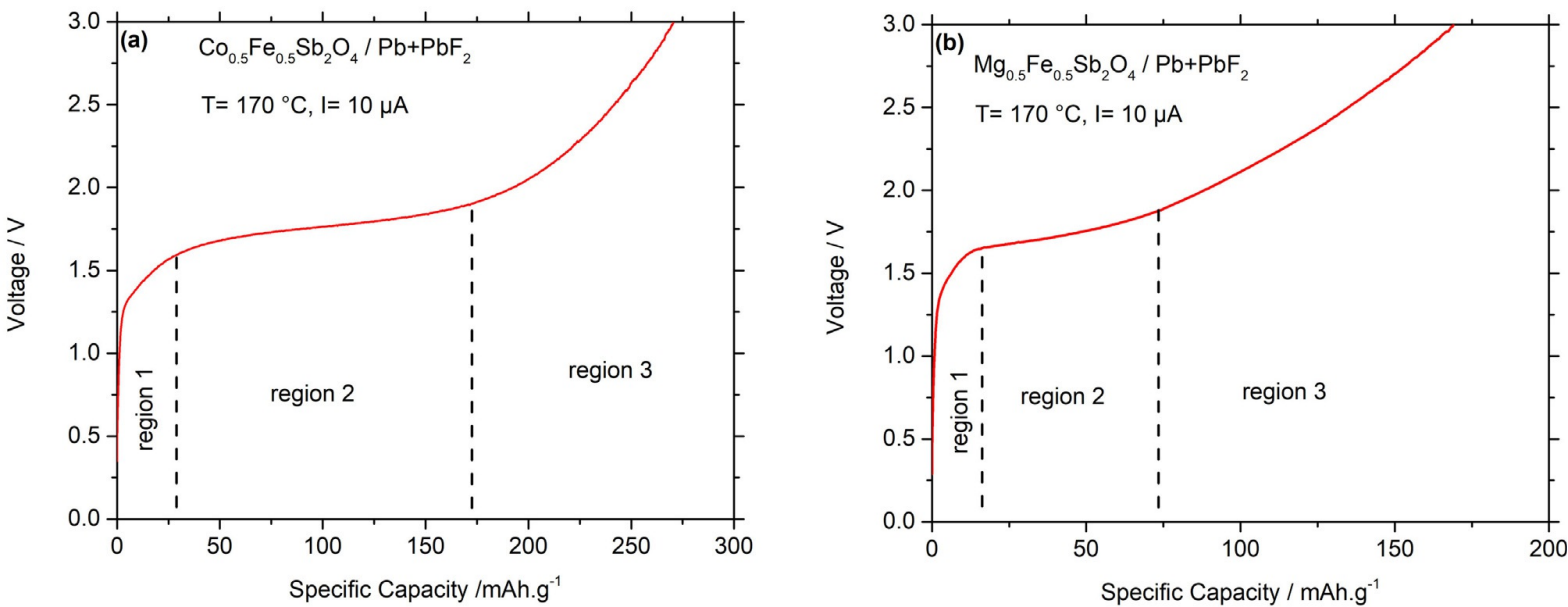
Electrochemical charging curves of a) Co_0.5_Fe_0.5_Sb_2_O_4_ and b) Mg_0.5_Fe_0.5_Sb_2_O_4_ at *T*=170 *°*C, *I*=10 μA (24 μA cm^−2^).

The electrochemical charging curves of Mg_0.5_Fe_0.5_Sb_2_O_4_ and Co_0.5_Fe_0.5_Sb_2_O_4_ against Pb+PbF_2_ are shown in Figure 3. The charging curves show three distinct regions: first a sharp increase up to 1.5 V, followed by a plateau between roughly 1.6–1.7 V. In the third region, a sharp increase in voltage is observed, indicating the end of the electrochemical reaction. Within the first region, no reaction of the schafarzikite‐type compounds could be identified (also see later in this article), and structural changes were mainly found to occur in region 2.

Greaves and co‐workers6b reported a capacity of approximately 0.5 fluoride ions per formula unit Mg_0.5_Fe_0.5_Sb_2_O_4_ and Co_0.5_Fe_0.5_Sb_2_O_4_ via chemical fluorination, which would correspond to capacities of 36–39 mAh g^−1^ for the charging/electrochemical oxidation reaction corresponding to [Eq. (2)]:

(2) $$\mathrm{MSb}{}_{2}O{}_{4}+0.5\;F{}^{-}\to \mathrm{MSb}{}_{2}O{}_{4}F{}_{0.5}+0.5\;e{}^{-}$$

The lengths of the observed charging plateaus in region 2 exceed this capacity significantly, which can be explained from an overlap of the charging plateau with the electrochemical fluorination of the conductive additive of carbon to C−F species^16^ (the amount of carbon added can contribute to a charging capacity which is at least 10 times higher than the absolute capacity originating from the amount of the schafarzikite compounds9a). As found previously, this can impede the discharging (defluorination) of the target compounds, owing to the destruction of the electronic conductive matrix under formation of C−F species.^16^ This would prohibit their use as cathode materials for reversible fluoride‐ion batteries9a when carbon is used as an additive for achieving electronic conductivity within the active composite. For Co_0.5_Fe_0.5_Sb_2_O_4_, the plateau region is significantly longer, which might be explained by a higher catalytic activity for the fluorination of carbon. In contrast, the decomposition of the electrolyte at the cathode side can be basically ruled out, as La_0.9_Ba_0.1_F_2.9_ is not sensitive towards oxidation, as verified by no significant changes of the cell parameters of the solid electrolyte after charging (see Table S1).

Furthermore, we note that the potential for the fluorination of the schafarzikite compounds, which only involves the Fe^2+^/Fe^3+^ redox couple,6b is higher than Mn^3+^/Mn^4+^ and Co^2+^/Co^3+^ in LaSrMnO_4_ and La_2_CoO_4_, respectively (ca. 1.2 V for LaSrMnO_4_ and 0.9 V for La_2_CoO_4_ against a composite of Pb+PbF_2_ at the same condition),^8^, ^9^ and this would not be expected intuitively from the electrochemical series.^17^ This could either result from an unusually high electrochemical potential of Fe^2+^ within this structure type, or from higher overpotentials for the schafarzikite‐type structure as compared to the Ruddlesden–Popper‐type compounds. This might be related to a different dimensionality of the fluoride‐ion sublattices [channels (1D) of fluoride ions in MSb_2_O_4_ vs. planes (2D) of fluoride ions in A_2_MO_4_ (A=Sr, La)]. A comparison of the charging and discharging plateau of these materials is provided in Figure S1.

The change in lattice parameters after electrochemical fluorination can be followed visually from the changes of the reflection positions in Figures 2 c and 2 d, where the refined values given in Table 1 are consistent with the changes found by Greaves and co‐workers.6b The small difference in the lattice parameters between the chemically fluorinated and electrochemically fluorinated samples (please see Table 1) could arise from slightly different amounts of intercalated fluorine within each sample. In our previous article,^8^ different cut‐off capacities in combination with a quantitative phase analysis of the fluorinated and non‐fluorinated phase were used to determine the detailed amount of intercalated fluoride ions. However, such attempts to investigate the fluorination process intercalation process in more detail by choosing different cut‐off capacities did not prove to be successful in this study; here, we either observed the lattice parameters of the unreacted starting product, or the lattice parameter had changed within errors to the ones obtained after charging to 3 V. No non‐fluorinated parent phase was found in addition to the fluorinated phase, and this is different to the electrochemical charging of La_2_CoO_4_ to La_2_CoO_4_F_1.2_, for which a coexistence of both phases can be found in the plateau region. Therefore, the fluorination of schafarzikite‐type compounds to compounds with the composition MSb_2_O_4_F_*x*_ appears to result in single‐phase compounds for a broad region of *x*, whereas compositions La_2_CoO_4_F_*x*_ (0<*x*<1.2) appear to result in two‐phase mixtures of (^*x*^/_1.2_ La_2_CoO_4_F_1.2_ + ^(1.2−*x*)^/_1.2_ La_2_CoO_4_).

We also note that the cell parameters after the fluorination possess a pseudocubic metric [*a*/(c*√2)] ≈1, see Table 1). This ratio is well reproduced regarding the chemical and electrochemical fluorination processes. We investigated the possibility that a higher cubic symmetry could exist for fluorinated compounds by testing possible supergroups of the tetragonal schafarzikite‐type structure. However, a rearrangement of polyhedra within the schafarzikite structure to result in a three‐fold rotational axis (required for cubic symmetry) does not appear possible. Therefore, no simple group–subgroup relationships could be identified, which would explain a change to cubic symmetry, and this is in agreement with previous symmetry analyses.^8^

The volume changes of the active cathode material structures also calculated to be approximately 1.8 and 1.7 % for Co_0.5_Fe_0.5_Sb_2_O_4_ and Mg_0.5_Fe_0.5_Sb_2_O_4_, respectively (from 433.8(2) to 426.2(4) Å^3^ on fluorination for Co_0.5_Fe_0.5_Sb_2_O_4_, and from 433.0(2) to 425.7(2) Å^3^ on fluorination for Mg_0.5_Fe_0.5_Sb_2_O_4_. We would like to point out that those changes are very low as compared to Ruddlesden–Popper‐type compounds, which are on the order of 10–20 %.^5^, ^8^, ^9^

**Figure 4:**
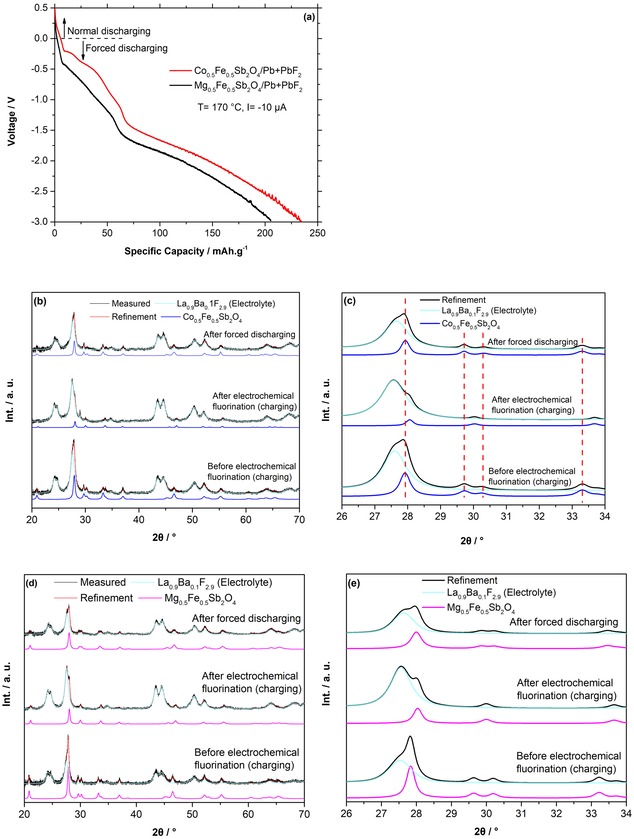
a) Forced discharging of Co_0.5_Fe_0.5_Sb_2_O_4_ and Mg_0.5_Fe_0.5_Sb_2_O_4_ against Pb+PbF_2_ at *T*=170 *°C*, *I*=10 μA (24 μA cm^−2^). Respective XRD measurement after forced discharging of the b, c) Co_0.5_Fe_0.5_Sb_2_O_4_ and d, e) Mg_0.5_Fe_0.5_Sb_2_O_4_ cells.

Once fully charged, the discharge profiles of the materials were investigated, as shown in Figure 4 a. The discharge capacities were found to be very low, on the order of 6.0 mAh g^−1^ (corresponding to ca. 0.08 F^−^) and 3.0 mAh g^−1^ (corresponding to ca. 0.04 F^−^) for Co_0.5_Fe_0.5_Sb_2_O_4_ and Mg_0.5_Fe_0.5_Sb_2_O_4_, respectively (see Figure 4 a). This observation is similar to our previous findings for LaSrMnO_4,_ 9a for which the charging plateau also was found to overlap with the decomposition of the carbon matrix. However, on discharging to negative potentials against Pb/PbF_2_ (“forced discharging” due to accessing potentials that would correspond to an endergonic or hindered process, as would be the case for the destruction of carbon on charging), lattice parameters were found to change back to close to the values observed for the starting materials (see Table 1 and Figure 4 b, c). For Mg_0.5_Fe_0.5_Sb_2_O_4_, the difference in lattice parameters compared to the unreacted compound (Δ*a*=0.032 Å and Δ*c*_MFSO_=0.011 Å) is bigger (although overall small) than that for Co_0.5_Fe_0.5_Sb_2_O_4_ (Δ*a*_CFSO_=0.002 Å and Δ*c*_CFSO_=0.010 Å), which might indicate the presence of residual fluoride ions within the compound (see Table 1 and Figure 4 d, e). From the shape of the discharging curve, one can also derive a principle fluorine content in the order of 40–50 mAh/ g, which corresponds to 0.5–0.6 fluoride ions and is in well agreement with the fluorine contents found for the chemical fluorination reactions.6b

These findings show that the schafarzikite‐type structure allows for reversible intercalation/deintercalation of fluoride ions through electrochemical fluorination, making it the second structure type known for the structurally reversible incorporation of fluoride ions so far. As with the fluorinated Ruddlesden–Popper‐type compounds,^8^, 9a the oxide and fluoride in the fluorinated schafarzikite structure have been shown6b to occupy two different crystallographic sites, see Figure 1. These sites were determined from neutron powder diffraction studies, where the bond distances from the structural solutions were used to calculate bond valences sums to support the validity of the proposed models. Furthermore, it was suggested that the fluoride ions only form bonds to the soft antimony cations (and not to the transition metal M) without primarily oxidizing the Sb^3+^ to Sb^5+^. Such bonding behavior and associated localized structural distortions could lower the activation energy of fluoride ions for migration through the structure. Compounds with ns^2^ cations (such as SnF_2_, PbF_2_, and SbF_3_)^18^ are known to be good fluoride‐ion conductors, owing to the high polarizability of the cations. Therefore, the local chemical environment of the fluoride ions in the schafarzikite structure closely resembles the situation found in the binary fluorides of ns^2^ metals. Again, this also resembles the scenario found in the Ruddlesden–Popper‐type structure, where fluoride ions only form bonds to the alkaline‐earth/rare‐earth cations (for which the binary metal fluorides are also good fluoride‐ion conductors).^18^ Both structural features (anion ordering and type of M−F bonds formed) might, therefore, determine a prerequisite for the selective deintercalation of fluoride ions and full structural reversibility.

## 3 Conclusions

In this article, we have shown that the electrochemical fluorination process is applicable to schafarzikite‐type compounds Mg_0.5_Fe_0.5_Sb_2_O_4_ and Co_0.5_Fe_0.5_Sb_2_O_4_. Analysis of lattice parameters before electrochemical fluorination and after charging/discharging revealed a close similarity of products for both reaction routes. This shows that fluorinated schafarzikite compounds can be prepared by using significantly milder, less dangerous reaction conditions through electrochemical routes. However, it should be taken into consideration that the final fluorinated product is mixed with the electrolyte material and carbon additive and, so far, no separation strategies were examined to obtain the electrochemical products isolated from the additives; furthermore, the material is obtained in low quantity compared to what can be obtained by using chemical methods. The voltage plateau of the intercalation process coincides with the decomposition of the conductive additive carbon, which currently makes the material a bad candidate for battery applications unless other more stable conductive additives can be found (the authors would like to point out that such attempts were made, for example, by using silver, but did not prove to be successful). Regardless of this, the compounds show excellent structural reversibility for the fluorine intercalation/deintercalation process, which is most likely facilitated by the ordering of oxide and fluoride ions in addition to local bonding scenarios around the fluoride ions and their arrangement within 1D channels. In the future, we aim to extend our investigation to other schafarzikite compounds or compounds within the Mullite family.^19^

## Experimental Section

Schafarzikite‐type compounds with the composition Mg_0.5_Fe_0.5_Sb_2_O_4_ and Co_0.5_Fe_0.5_Sb_2_O_4_ have been prepared by using the method described by de Laune et al.6b Stoichiometric amounts of a dried mixture of the metal oxides and antimony metal (CoO, 325 mesh Sigma–Aldrich; Fe_2_O_3_, ≥99.9 % Sigma–Aldrich; Sb_2_O_3_, Reagent Plus, Sigma–Aldrich; Sb, BDH; MgO, ≥99 % 325 mesh Sigma–Aldrich) were heated in evacuated sealed quartz tubes for between 6 and 36 h at 700 °C, with intermittent grinding.

An electrochemically active composite (EAC) was prepared by mixing the Co_0.5_Fe_0.5_Sb_2_O_4_ and Mg_0.5_Fe_0.5_Sb_2_O_4_ compounds with La_0.9_Ba_0.1_F_2.9_ (a fluoride‐conducting electrolyte,^20^ in accordance with previous studies)9a and dried black carbon in a weight ratio of 30:60:10, respectively. The mixture was milled for 3 h at a rotational speed of 250 rpm (Retsch PM100‐CM, for 10 min intervals with 20 min of resting between the intervals). The volume of the milling vial and the diameter of each ball were approximately 244 cm^−2^ (0.24 L) and 10 mm, respectively. The ball‐to‐powder ratio was 30:1 using 10 balls with a total mass of almost 30 g. All milling processes were performed in ZrO_2_ vials, which were filled and sealed inside a high‐purity Ar‐filled (99.999 %) glovebox. A composite of Pb+PbF_2_, as previously described in Ref. 9a was used as the counter electrode and the source of fluoride ions. The use of the EAC instead of pure schafarzikite compounds is required, owing to the insufficient fluoride‐ion and electronic conductivity of pure schafarzikite at low temperatures, and this is a common procedure for the investigation of electrode compounds.4a

For electrochemical fluorination/defluorination, a fluoride‐ion battery setup was used.9a Three layers (EAC, La_0.9_Ba_0.1_F_2.9_, and Pb+PbF_2_) were compacted to a battery cell at a load of 2 tons for 90 s over an area of 0.42 cm^2^, using a desktop press (Specac) and steel die set inside an Ar‐filled glovebox. The dimensions of the overall cell were measured to be 1.6 mm thick and 7.3 mm in diameter. Battery cells were spring‐loaded (as described in Ref. 9a) into a modified Swagelok‐type cell with current collectors made of stainless steel. The applied charging and discharging currents were chosen to be ±10 μA (24 μA cm^−2^). The values of the charging/discharging current are based on our previous experience on the magnitude of overpotentials during the charging/discharging reactions.9a To ensure sufficient mobility of the fluoride ions, the electrochemical cells were heated by band heaters and measurements were taken at 170 °C. The temperature of 170 °C was chosen, as it facilitates sufficient conductivity of the solid electrolyte, which is required to limit overpotentials arising from the so‐called *IR* drop to below 0.1 V for the current densities used in this study (below 24 μA cm^−2^).^20^, ^21^ A potentiostat (BioLogic SP‐150 & VSP300) was used for all of the galvanostatic charging measurements.

Ex situ X‐ray diffraction was used to monitor structural changes of the target compounds. The measurements were performed by using a Bruker D8 Advance in Bragg–Brentano geometry and Cu K_*α*_ radiation (VANTEC detector). To avoid potential side reactions with the atmosphere, all samples were loaded into a low‐background specimen holder (Bruker A100B36/B37) and sealed inside an Ar‐filled glovebox before every measurement. Data were recorded between 20 and 70° (2*θ*) for a total measurement time of 4 h using a step size of approximately 0.007° and a fixed divergence slit of 0.3°. All analyses of diffraction data were performed by using the Rietveld method in TOPAS V5.^22^ The instrumental intensity distribution, that is, the apparative broadening of reflections, was determined empirically from a fundamental parameter set by using a reference scan of LaB_6_ (NIST 660a). The microstructural parameters (crystallite size and strain broadening) were refined to adjust the peak shapes. Thermal displacement parameters were refined and constrained to be the same for all of atoms of all phases to minimize quantification errors and to account for angular dependent intensity changes induced by absorption and surface roughness.

## Conflict of interest

*The authors declare no conflict of interest*.

## Supporting information

As a service to our authors and readers, this journal provides supporting information supplied by the authors. Such materials are peer reviewed and may be re‐organized for online delivery, but are not copy‐edited or typeset. Technical support issues arising from supporting information (other than missing files) should be addressed to the authors.

Supplementary
