# Supplementary material for: Reversible Electrochemical Intercalation and Deintercalation of Fluoride Ions into Host Lattices with Schafarzikite‐Type Structure
Source: ChemistryOpen. 2018 Aug 20;7(8):617–23. doi: 10.1002/open.201800106 (PMC6099171; doi:10.1002/open.201800106)
Supplement: Supplementary file 1 — Supplementary [file OPEN-7-617-s001.pdf]

ChemistryOPEN

Supporting Information

© 2018 The Authors. Published by Wiley-VCH Verlag GmbH & Co. KGaA, Weinheim

Reversible Electrochemical Intercalation and Deintercalation of Fluoride Ions into Host Lattices with Schafarzikite-Type Structure

Mohammad Ali Nowroozi,[a] Benjamin de Laune,[b] and Oliver Clemens*[a, b, c]

open_201800106_sm_miscellaneous_information.pdf

Comparison of charging and discharging behaviour to other cathode materials reported previously

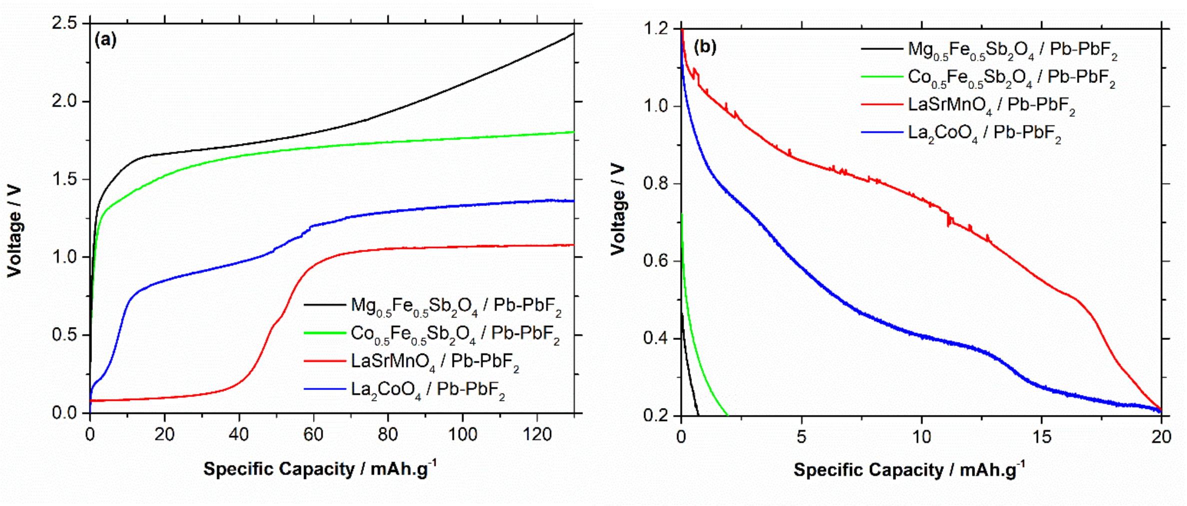

Figure S 1. $LaSrMnO_4$ [1], La_2CoO_4 [2], $Co_{0.5}Fe_{0.5}Sb_2O_4$, and $Mg_{0.5}Fe_{0.5}Sb_2O_4$ against Pb-PbF_2 anode material at 170 °C, I_{charge}= 10 µA (24 µA/cm^2) and $I_{discharge}$= -1.0 A (-2.4 µA/cm^2) (a) electrochemical fluorination (charge); (b) electrochemical de-fluorination (discharge).

Table S 1. Lattice parameters of the $La_{0.9}Ba_{0.1}F_{2.9}$ electrolyte material before and after various electrochemical treatments or heating.

	$La_{0.9}Ba_{0.1}F_{2.9}$ within $Co_{0.5}Fe_{0.5}Sb_2O_4$ cathode material			$La_{0.9}Ba_{0.1}F_{2.9}$ within $Mg_{0.5}Fe_{0.5}Sb_2O_4$ cathode material		
	Crystal structure / Space group	a [Å]	c [Å]	Crystal structure / Space group	a [Å]	c [Å]
**Initial material (before mixing with the cathode material)**	Trigonal/*P*-3*c*1	7.2257(24)	7.3908(27)	Trigonal/*P*-3*c*1	7.2257(24)	7.3908(27)
**As mixed before heating)**	Trigonal/*P*-3*c*1	7.2349(10)	7.4054(13)	Trigonal/*P*-3*c*1	7.2268(13)	7.3967(18)
**After heating**	Trigonal/*P*-3*c*1	7.2280(6)	7.3996(8)	Trigonal/*P*-3*c*1	7.2248(11)	7.3941(15)
**After electrochemical fluorination (charging)**	Trigonal/*P*-3*c*1	7.2315(8)	7.4006(9)	Trigonal/*P*-3*c*1	7.2244(7)	7.3935(8)
**After electrochemical de-fluorination**	Trigonal/*P*-3*c*1	7.2171(10)	7.4065(13)	Trigonal/*P*-3*c*1	7.2224(9)	7.4008(11)

References

[1] M. A. Nowroozi, K. Wissel, J. Rohrer, A. R. Munnangi and O. Clemens, *Chem. Mater.* **2017**, *29*, 3441-3453.
[2] M. A. Nowroozi, S. Ivlev, J. Rohrer and O. Clemens, *J. Mater. Chem. A* **2018**, *6*, 4658-4669.
